# An incremental anomaly detection model for virtual machines

**DOI:** 10.1371/journal.pone.0187488

**Published:** 2017-11-08

**Authors:** Hancui Zhang, Shuyu Chen, Jun Liu, Zhen Zhou, Tianshu Wu

**Affiliations:** 1 College of Software Engineering, Chongqing University, Chongqing, China; 2 College of Computer Science, Chongqing University, Chongqing, China; 3 College of Software Engineering, Chongqing University of Posts and Telecommunications, Chongqing, China; 4 School of computer science and technology, Southwest Minzu University, Chengdu, China; Southwest University, CHINA

## Abstract

Self-Organizing Map (SOM) algorithm as an unsupervised learning method has been applied in anomaly detection due to its capabilities of self-organizing and automatic anomaly prediction. However, because of the algorithm is initialized in random, it takes a long time to train a detection model. Besides, the Cloud platforms with large scale virtual machines are prone to performance anomalies due to their high dynamic and resource sharing characters, which makes the algorithm present a low accuracy and a low scalability. To address these problems, an Improved Incremental Self-Organizing Map (IISOM) model is proposed for anomaly detection of virtual machines. In this model, a heuristic-based initialization algorithm and a Weighted Euclidean Distance (WED) algorithm are introduced into SOM to speed up the training process and improve model quality. Meanwhile, a neighborhood-based searching algorithm is presented to accelerate the detection time by taking into account the large scale and high dynamic features of virtual machines on cloud platform. To demonstrate the effectiveness, experiments on a common benchmark KDD Cup dataset and a real dataset have been performed. Results suggest that IISOM has advantages in accuracy and convergence velocity of anomaly detection for virtual machines on cloud platform.

## Introduction

As cloud computing continues to develop, cloud platform based on virtualization technology is becoming increasingly popular in the fields of medicine, biology, geology and scientific computing and so on. The scale of virtual machines in cloud platform is continuously growing, and the applications deployed on virtual machines are more and more complex. At the same time, competition for resources in the cloud platform, resource sharing, virtual machine overload are prone to cause abnormalities which will make a part of the virtual machines downtime and will affect the reliability and availability of the entire cloud platform seriously. Therefore, it is highly desirable to provide an effective anomaly detection algorithm for virtual machines in cloud platform [1–4].

Nowadays, statistics, clustering, classification, and nearest-neighbor algorithms are commonly used methods of detecting abnormal [5]. Method under a certain probability model is based on statistical anomaly detection algorithm, nevertheless, a hypothetical probability model has to be predefined [6]. SVM-based [7] algorithms, Bayes networks [8] and neural networks [9] are the main several classification algorithms for anomaly detection used to learn a classify model (classifier) from the training data (labeled data instances) and then to predict a test instance using the classifier. Here, classification-based techniques rely on various labeled instances, which is hard to get and be labeled in real problems. Moreover, for multi-class classification, to get a variety of labeled normal classes is almost impossible. Clustering is an unsupervised learning method, which can find groupings in input instances without any prior knowledge [10–12]. Therefore, comparatively, clustering is a useful tool in dealing with a large, complex and unforeseen dataset with many variables and unknown structures.

Self-Organizing Map (SOM) algorithm is a clustering and data analysis algorithm proposed by Kohonen in 1982 [13, 14]. Since that time, it has been widely used and the improvement of the algorithm has been going on. In order to solve the initialization problem of the size of SOM network, Growing SOM algorithm [15]has been proposed. The main idea is to form a single n-dimensional space (e.g. triangle, tetrahedron) as the basic building blocks in the training process of generating dynamic SOM network. Meanwhile, for the purpose of achieving SOM algorithm rapidly, TS-SOM (Tree Structured Sel-Organizing Maps) [16–18] method was proposed to realize the SOM by using the recursive nature of the tree structure itself. Furthermore, in order to address the problem of SOM network size and parameter optimization, Polani [19], Kubota R [20], and Deep K [21]et al. applied Genetic Algorithm to evolve SOM network to figure out the fitness function which optimized SOM network, to get the optimal solution of the initial size of SOM network, and to train the training neighborhood of the SOM network by the optimized function. Due to the characteristics of non-supervisory, non-linear, lower complexity and mapping high-dimensional data into low-dimensional space, SOM has been widely used in pattern recognition [22, 23], biology [24, 25], medical applications [26], climate [27, 28], neural networks [29, 30], image analysis [31] and other fields.

However, the application of SOM in anomaly detection for virtual machine is still in its infancy. Hoz E D L [32] et al. presented a Growing Hierarchical SOM (GHSOM) for network intrusion detection on the basis of NSGA-II algorithm for feature selection to reduce the complexity of SOM. A new SOM map arose or not in GHSOM need to be calculated with the quantization error of each unit. Although it reduced the computational complexity of dataset dimension as a result of feature selection, a lot of calculations and comparisons were still required and the training time increase. In addition, they proposed a Probabilistic SOM (PSOM) approach that hybridizes statistical technique and SOM for network intrusion detection where Principal Component Analysis (PCA) and Fisher Discriminant Ratio (FDR) were considered for feature selection and noise removal [33]. In PSOM, the SOM map was trained by a Gaussian Mixture Model (GMM) first, while a further tuning of the map was achieved by calculating the prior probabilities and posterior probability of the SOM units. Consequently, PSOM would spend a lot of training time and computing costs on the input samples to achieve a good classification result. When in case of the large scale and high dynamic of virtual machines under the cloud platform, they would appear a high false positive rate and a low accuracy rate. Jun L [34] et al. applied Self-Organizing Maps method for anomaly detection of cloud platform and presented a unified modeling method based on SOM with a detection region. However, they did not optimize the SOM algorithm and it significant affected the accuracy and speed of detection. Wang H [35] et al. proposed a Simulated Annealing-based SOM (SOMSA) method for intrusion detection. The Simulated Annealing (SA) algorithm was used to refine the weight of SOM to get the optimal point by a form of probability. Nevertheless, SA is a random search algorithm. In each process of refining, the chose of best matched neuron and its neighborhoods is performed in random with probability. As a result, this method achieved the accuracy at the cost of training time due to the characteristic of random search. Based on the process units of anomaly detection, Song Y [36] et al. proposed a Statistic Pattern-based SOM (SP-based SOM) method, which had advantages on a small-scale system. Whereas, because of the feature of complex and dynamic of cloud platform and a large amounts of performance metrics, this method was not suitable for cloud platform with large-scale virtual machines. Moreover, Song Y et al. did not optimize the initialization and the training neighborhood of SOM, which significantly affects the speed of modeling SOM network.

To address these problems, this paper presents an Improved Incremental SOM (IISOM) anomaly detection algorithm for virtual machines on cloud platform, in which a heuristic SOM algorithm has been proposed to initialize SOM network and a Weighted Euclidean Distance (WED) method has been used to improve the training neighborhood. As mentioned above, Wang H [35] et al. utilized the Simulated Annealing-based SOM algorithm to obtain global optimal solution, which has advantages in improving the accuracy of anomaly detection for virtual machines on cloud platform. But due to the expense of training time, it is not functional for anomaly detection of virtual machines with high dynamic. Here, in order to obtain a global optimal solution and reduce the training time, IISOM introduces the heuristic-based initialization algorithm to consider the similarity of input data instance and the characteristics of SOM convergence to estimate the weight of SOM, which has the advantages in global optimal clustering the same as SOMSA. Moreover, IISOM is superior to SOMSA subjected to detection accuracy and convergence rate as well as the quality of model. The effectiveness of the proposed method can be substantiated by systematic analysis associated with the benchmark dataset KDD Cup and real dataset. It can be found that the IISOM has a much higher training speed and accuracy than the traditional SOM algorithm and SOMSA algorithm.

The rest of this paper is organized as follows. In Materials and Methods, the traditional SOM algorithm and the SOMSA algorithm are described. And according to the lacks of traditional SOM and SOMSA method applied for anomaly detection of virtual machines in cloud platform, the optimistic IISOM method is proposed. Then carry out the experiments to show the performance evaluation. Advantages and limitations of the IISOM are discussed in the discussion part and also we give some expectations for the future work. And finally, conclude this paper.

## Materials and methods

### Traditional SOM algorithm

Self-Organizing Map (SOM) is a popular neural network model that using unsupervised learning rules to analyze, cluster, and model various datasets. Commonly, it is used to map a high dimension input space into a low dimensional discrete map space. Meanwhile it preserves the topological properties of the original input space [37, 38].

**Fig 1:**
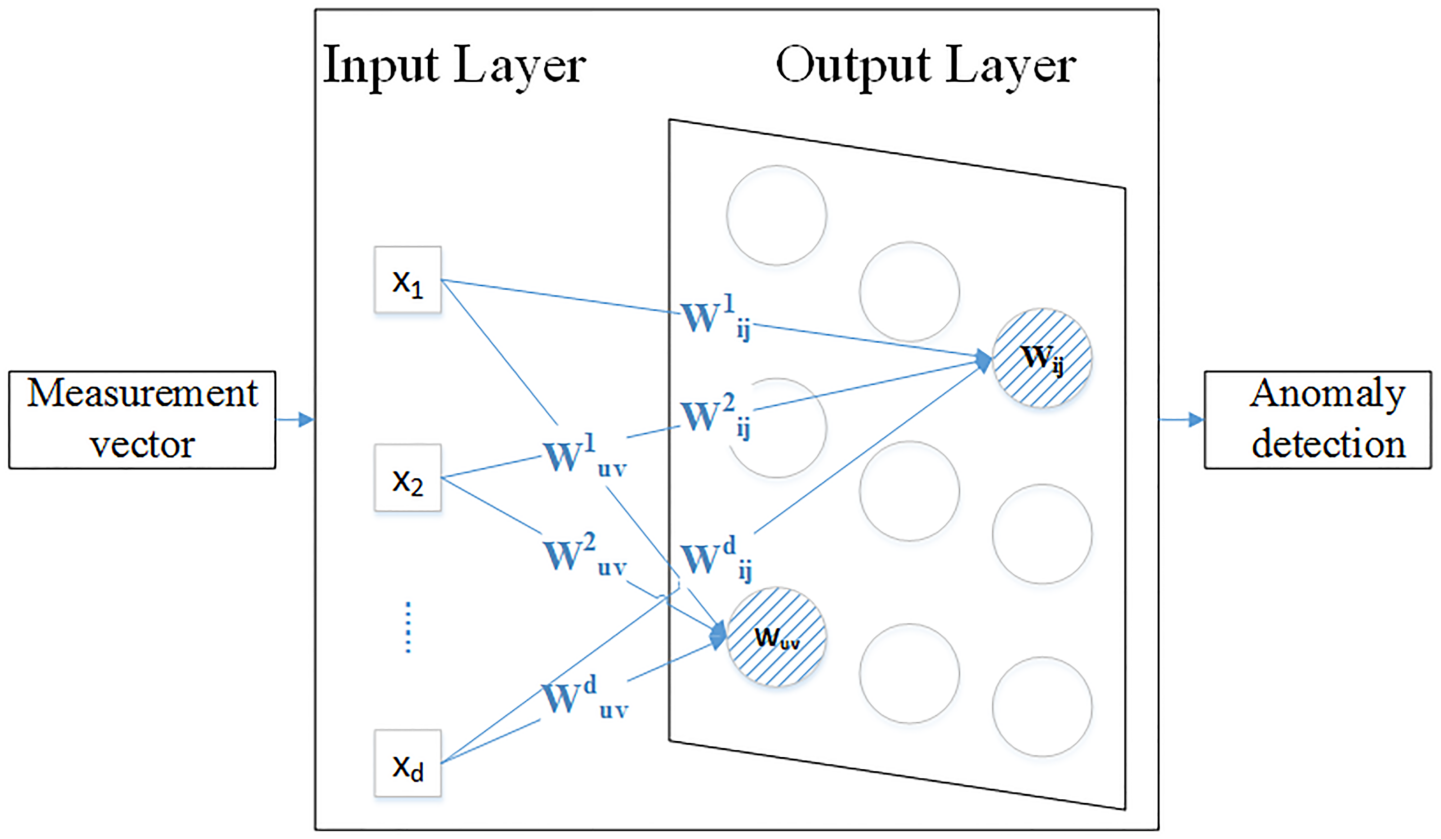
The general structure SOM network. SOM network consists of the Input Layer and Output Layer. Each input vector X is a D-dimensional vector, and each neurons in the output layer is associated with a coordinate(u, v) and a weight vector W_*uv*_.

Generally, SOM consists of two layers: the input layer, denoting the runtime measurement vectors, and the output layer, usually consisting of a two-dimensional lattice type of neurons, illustrated by Fig 1. The training of SOM network is usually done in two phases: ordering or self-organizing phase to get a rough training order and then convergence phase to fine-tune the map and to provide the ability for detection. Here we give a brief introduction.

Suppose the input vector of runtime measurements is x(t) = [x_1_, x_2_, x_3_, …, x_*d*_] ∈ R^*D*^, and there are N*N neurons in the output space, a two-dimensional lattice network, in which each neuron n_*uv*_ is associated with a coordinate(u, v), 1 ≤ u, v ≤ N and a weight vector W_*uv*_. Here, x_*h*_ (1≤ h ≤ D)denotes one system-level performance metrics, such as CPU, memory, disk I/O, or network traffic and so on, and the weight vector W_*uv*_ should have the same length as the measurement vector x(t).

**Fig 2:**
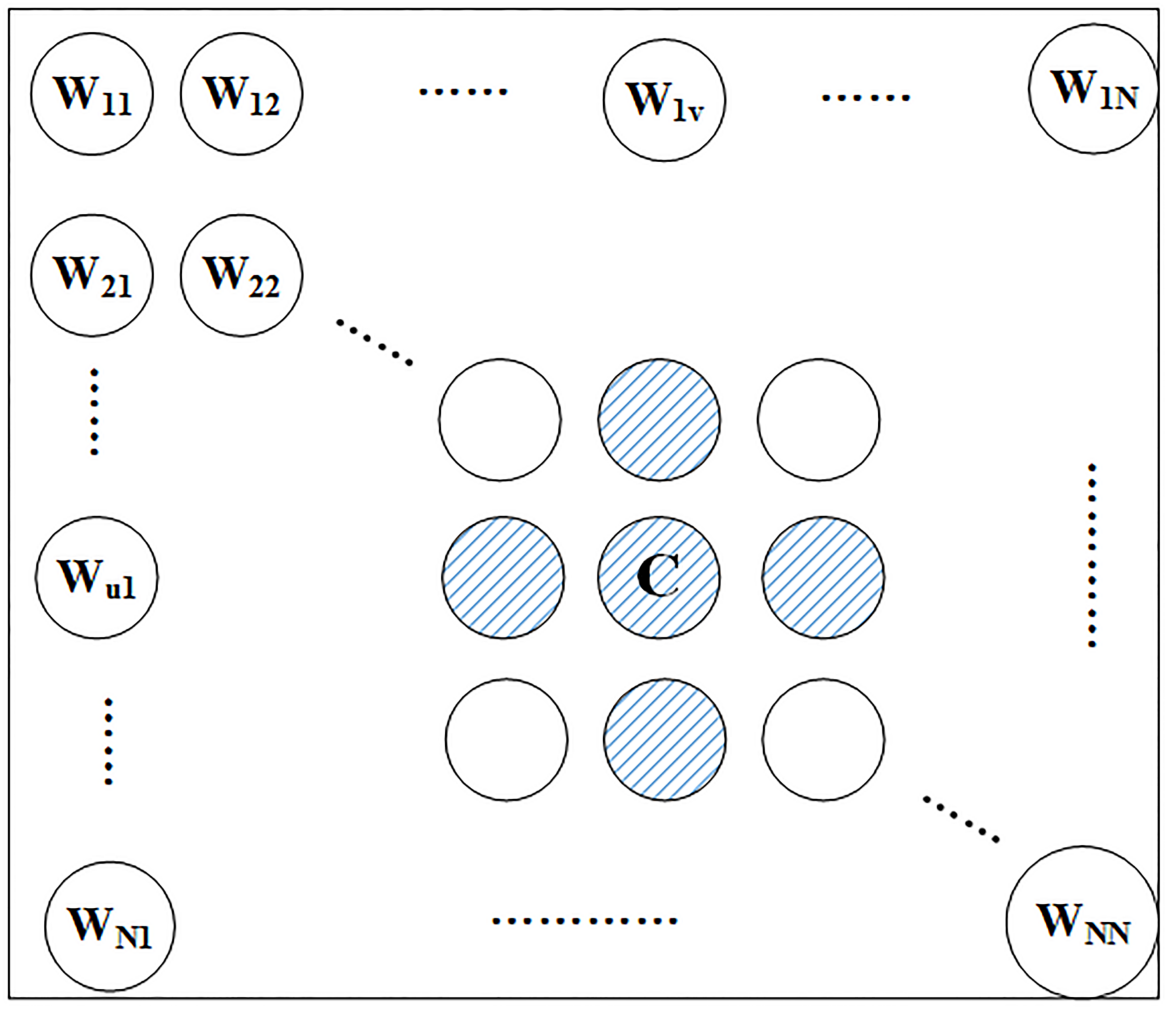
The nearest neighbors of excited neuron C. Each neuron is labeled with it’s coordinate (u, v), that is W_*uv*_. C: the excited neuron. The circles with blue line is the nearest neighbors of excited neuron C.

The SOM network training process is the neuron competition process, which works by computing the distances between the input measurement vector and each neuron’s weight vector in the map and selecting the neuron with the smallest distance as the winning neuron or excited neuron. Several choices can be made for the definition of the distance function such as Euclidean distance, Manhattan distance, Cosine similarity etc. And then the winning neuron’s weight vector as well as its neighborhood neurons are updated as shown in Fig 2. The basic formula for updating the weight vector of a given neuron (u, v) at time t is given in Eq (1).

$$\begin{array}{c} W_{uv}\left(t\right)=W_{uv}\left(t-1\right)+N_{C}\left[x\left(t\right)-W_{uv}\left(t-1\right)\right] \end{array}$$ (1)

Where W_*uv*_(t-1) is the weight vector at time t-1, and x(t) is the input vector of system-lever performance metrics at time t. N_*C*_ is a neighborhood function of the winning neuron or excited neuron C, and a Gaussian function is usually used as the neighborhood function. The function is described in Eq (2).

$$\begin{array}{c} N_{C}\left(t\right)=\gamma \left(t\right)\times exp\left(-\frac{∥I_{C}{-\left(i,j\right)∥}^{2}}{2\delta^{2}\left(t\right)}\right) \end{array}$$ (2)

Where N_*C*_ is the neighborhood of the excited neuron C, *γ*(t) is the learning-rate factor at time t, which determines how much each weight vector changed at time t, I_*C*_ is the index of the excited neuron C, (i, j) is the coordinate of neuron n_*ij*_ in the map, *δ*(t) is the size of the neighborhood at time t. *γ*(t) and *δ*(t) is monotonically decreasing with time t.

The winning neuron or excited neuron is determined by the squared Euclidean distance between the input vector x(t) and the weight vector W_*uv*_, for each input vector the excited neuron (u, v) is determined in Eq (3).

$$\begin{array}{c} C=\{\begin{array}{cc} \arg\min_{uv}∥x\left(t\right)-W_{uv}\left(0\right)∥, & t=1\\ \arg\min_{uv}∥x\left(t\right)-W_{uv}\left(t-1\right), & t=2,3,\ldots \end{array} \end{array}$$ (3)

To determine whether the training process of SOM network is convergence, the following inequality Formula Eq (4) is used.

$$\begin{array}{c} \frac{1}{N\times N}\sum_{i,j=1}^{N}∥W_{ij}\left(t\right)-W_{ij}\left(t-1\right)∥\leq \varepsilon \end{array}$$ (4)

Where *ε* is a sufficient small number we predefined, which denotes the average deviation of the SOM weight vector at time t and t-1, ‖ • ‖ is the Euclidean distance between two vectors.

### Simulated annealing-based SOM algorithm

Simulated Annealing-based SOM (SOMSA) algorithm applies Simulated Annealing algorithm to SOM to optimize the training process, which could be divided into two steps. First of all, use traditional SOM algorithm to train input samples to get the Best Matching Neurons (BMN) or winner neurons and their neighborhoods. Secondly, use simulated annealing (SA) algorithm to adjust the weight of BMN and its neighborhoods to find the global optimization solution (clusters).

Simulated Annealing (SA) [39] is an optimization algorithm to find a global minimum of a problem among many local minimal, in which containing a solution space with a set of all possible solutions (clusters) and an objective function.

In SOMSA, the BMNs and their neighborhoods are regarded as solutions S, and the inter-class total distance as the objective function J. The objective function of SOMSA is described in Eq (5).

$$\begin{array}{c} J_{S}=\sum_{i}^{m}\sum_{X\in S_{i}}∥X-C_{si}∥ \end{array}$$ (5)

Where X is the input vector, C_*si*_ is the center of cluster i in solution S, ‖ • ‖ is the Euclidean distance between the input sample X and its cluster center C_*si*_, J_*S*_ is the sum distance of each input sample to its cluster center.

Keep the SA algorithm going on until the optimal performance is achieved. At each iteration it replaces the current cluster center by a random nearby center chosen with a probability p, depending on the difference between the new objective function and the current objective function. If J_*S*′_-J_*S*_ is less than zero, then set S’ as the current optimal cluster solution, otherwise accept the new solution by the probability p. The formula is as Eq (6).

$$\begin{array}{c} p=exp(\frac{|J_{S}^{\prime }-J_{S}|}{k\times J_{S}}) \end{array}$$ (6)

Where k is a constant, J_*S*_ is the current objective function with a cluster center that the input sample belongs to, J_*S*′_ is the new objective function when the input sample chosen a random nearby cluster center.

During the training process, random initial weight W is needed to be set, which is the same as traditional SOM algorithm. Although the SOMSA algorithm optimizes the accuracy and can effectively prevent the local optimum through the probability function, it still costs more time to train SOM network due to the random initial value and the random selection strategies, which cannot detect performance anomalies of large-scale virtual machines on cloud platform in real-time, leading to a low scalability and adaptability. At each iteration more calculations and comparisons are required to performed simultaneously. Moreover, an annealing speed *α* and the iteration number N have to be predefined, too.

In order to address the problem, the following study attempts to improve the scalability, adaptability, accuracy and high convergence rate of SOM model by modeling an incremental SOM model that combines a heuristic-based initial optimization algorithm and a Weighted Euclidean Distance algorithm into SOM.

## An improved incremental SOM anomaly detection algorithm

### Incremental model-An iterative regression process based on heuristic initialization algorithm

Commonly, when using the SOM method to execute the anomaly detection, at least 1000 samples were needed to make the SOM map into a roughly order. However, under cloud platform, the virtual machines are deployed randomly and dynamically. It is not possible to obtain the performance metrics of virtual machines before deployment. So an incremental model SOM method or an iterative regression SOM is more suitable for anomaly detection of virtual machines on cloud platform.

The incremental SOM starts from the initialization optimization of the SOM network. The common method of SOM network initialization is called the random initialization which selects N samples from the input space randomly. N is the number of neurons in SOM, and it constitutes the initial associated weight vector of each neuron. Relevant research [40–43] indicates that although the initial associated weight vector is determined without any prior knowledge, the SOM network is still able to reach the ordering state after several training iterations. However, it obtains the ordering state at the cost of longer training time of SOM model training. In this paper, we proposed a heuristic-based initialization method for SOM network to effectively initialize the SOM network and shorten the training time.

By analyzing the convergence phase of SOM, it can be shown that, during the training period of a sample x, the weight of a neuron in SOM network will be tuned as long as the neighborhood size N_*C*_ is reduced to contain only one neuron that is the cluster center itself. The set of those samples are recorded as TS(W_*nv*_), and the equation can be described in Eq (7).

$$\begin{array}{c} W_{uv}\left(t+1\right)=W_{uv}\left(t\right)+\gamma \left(t\right)\left[x-W_{uv}\left(t\right)\right]\;\forall x\subset TS\left(W_{uv}\right) \end{array}$$ (7)

Assume that W_*uv*_ will eventually converges to ${\bar{W}}_{uv}$, then when there exists a sample x belongs to TS(W_*uv*_), it can be derived as Eq (8).

$$\begin{array}{c} \lim_{t\to \infty }W_{uv}\left(t+1\right)=\lim_{t\to \infty }W_{uv}\left(t\right)+\lim_{t\to \infty }\gamma \left(t\right)[x-W_{uv}\left(t\right)]\\ \Rightarrow {\bar{W}}_{uv}={\bar{W}}_{uv}+\lim_{t\to \infty }\gamma \left(t\right)[x-W_{uv}\left(t\right)]\\ ∵t\to \infty ,\lim_{t\to \infty }\gamma (t)>0\\ ∴x={\bar{W}}_{uv} \end{array}$$ (8)

Taking account of all the input samples that belong to TS(W_*uv*_), it can get the relations in Eq (9).

$$\begin{array}{c} E_{TS(W_{uv})}\left(x\right)={\bar{W}}_{uv}\\ \Rightarrow \frac{\int_{TS(W_{uv})}x\cdot p\left(x\right)}{\int_{TS(W_{uv})}p\left(x\right)}={\bar{W}}_{uv} \end{array}$$ (9)

where p(x) is the probability density function of the input space. It can be seen obviously that it’s hard to get the distribution and the probability density of the input space to calculate the weight of neuron W_*uv*_, but it can get the approximately estimate value of W_*uv*_ based on the input samples, which can be used as the idea initialization value of SOM network.

The heuristic-based initialization method is an iteration method by estimating the weight of neuron W_*uv*_ use the equation in Eq (10).

$$\begin{array}{c} W_{uv}=\frac{\sum_{h=1}^{L}n\left(x_{h}\right)\cdot x_{h}}{|C_{uv}|} \end{array}$$ (10)

Where | C_*uv*_ | denotes the number of training samples in the cluster C_*uv*_, L is the number of types of input vectors that in cluster C_*uv*_, n(x_*h*_) is the times of one kind of sample x_*h*_ occur in C_*uv*_, and $\left|{\text{C}}_{uv}\right|=\sum_{h=1}^{L}n\left({\text{x}}_{h}\right)$.

The convergence of the training process of the SOM initialization can be checked using Eq (11).

$$\begin{array}{c} \frac{1}{N\times N}\sum_{u=1}^{N}\sum_{v=1}^{N}∥W_{uv}-W_{uv}^{l}∥\leq \varepsilon \end{array}$$ (11)

where W_*uv*_^*l*^ is the last iteration weight value of neuron n_*uv*_, W_*uv*_ is the current weight value of n_*uv*_, ‖ • ‖ is the Euclidean distance between them, and *ε* is a sufficiently small real number.

During the heuristic-based initialization training process, just a small-scale number of samples are collected from the sample dataset. Instead of directly assigning the selected sample vector values to neurons, it trains each neuron by taking account of the number of samples in each cluster and the times one sample occur in the cluster, which makes the trained SOM network close to the final convergence state and improves the training speed efficiently.

### Weighted Euclidean Distance (WED)

Generally, in each iterative training process of SOM, it will compare the Euclidean distance of the input samples to each neurons’ weight vector in the map to determine the training center neuron or winning neuron C_*uv*_, then amend the weight vector of C_*uv*_ and its neighborhood neurons according to the neighborhood function. The Euclidean distance measures the distance between two vectors by the square deviations of each dimension of them, during which the variance value of each dimension is treated equally. Nevertheless, actually each dimension x_*i*_ of the input space vector x(t) = [x_1_, x_2_, x_3_, …, x_*d*_] ∈ R^*D*^ may subject to different distribution. And if the variance of one dimension x_*i*_ of the input vector is much larger than the others, it will cause the imbalance problem of the training center neuron to determine due to the single dimension plays a decisive role, adversely affect the quality of the SOM model.

To address the problem, the Weighted Euclidean Distance (WED) method is proposed to represent the contributions of each dimension in the competition process of SOM through calculating the weight of each dimension in the input vector in Eq (12).

$$\begin{array}{c} weight\left(x_{i}\right)=\frac{{\left(\frac{v(x_{i})}{sum_{v}}\right)}^{-1}}{{\left(\frac{\sum_{i=1}^{d}v\left(x_{i}\right)}{sum_{v}}\right)}^{-1}} \end{array}$$ (12)

Where v(x_*i*_) is the variance of each dimension in input space, sum_*v*_ is the sum of variance of each dimension, that is ${\text{sum}}_{v}=\sum_{i=1}^{d}\text{v}\left({\text{x}}_{i}\right)$.

According to the weight determined by Eq (12), we get the formula of WED as follow in Eq (13).

$$\begin{array}{c} WED\left(X,W\right)=\sqrt{\sum_{i=1}^{d}weight\left(x_{i}\right)\cdot {\left(x_{i}-W_{i}\right)}^{2}} \end{array}$$ (13)

### Neighborhood-based training domain searching algorithm

As we know, at the convergence phase, each iteration has few impact on the associated weight vector of neurons in the SOM. Meanwhile, system-level performance metrics values can be regarded as substantially stable during a very short time because of the local properties of the virtual machine. Therefore, reducing the search space during the SOM network training process can effectively lessen the search times, decrease the computational complexity, and shorten the training time. Then we combine the WED with neighbor-based searching algorithm together to train the central point of training domain to reduce the complexity of the search operations in the SOM network training process for determining the central point of the training domain.

**Fig 3:**
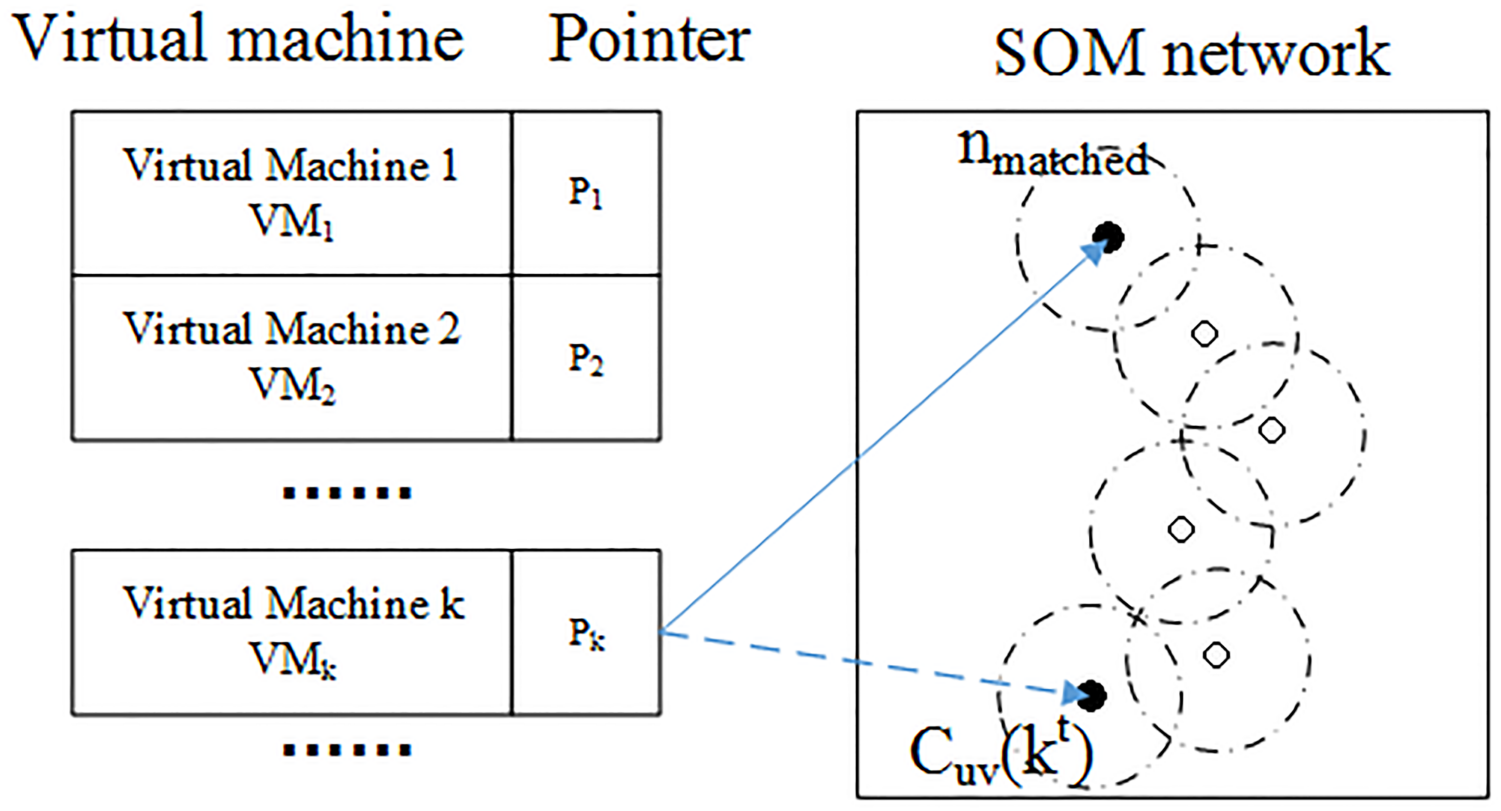
The process of neighborhood-based searching training domain algorithm. P_*k*_ is the point to virtual machine k, n_*matched*_ is the best matching neuron at time t + 1, and C_*uv*_(k^*t*^) is the excited neuron at time t.

The training process is shown in Fig 3. To describe the system-lever performance metrics of all the virtual machines (vm_*s*_) in the domain, let the token p_*k*_ as the pointer vector associated with virtual machine k (vm_*k*_), n_*matched*_ as the neuron that matches the current state, C_*uv*_(k^*t*^) as the central point of the training neighborhood determined by the runtime measurement samples of virtual machine vm_*k*_ at time t. So at time t, p_*k*_(t) points to C_*uv*_(k^*t*^). Assuming at time t + 1, p_*k*_(t + 1) still points to C_*uv*_(k^*t*^), then get the neighborhood of p_*k*_(t + 1) at time t + 1, which could be described as Left(p_*k*_(t + 1)), Right(p_*k*_(t + 1)), Top(p_*k*_(t + 1)), Down(p_*k*_(t + 1)). After that, using WED to compare the input vector vm_*k*_^*t*+1^ with C_*uv*_(k^*t*^) and its neighborhoods to find the best matching neuron n_*matched*_, which has the smallest Weighted Euclidian Distance. If n_*matched*_ ≠ C_*uv*_(k^*t*^), then change the pointer p_*k*_(t + 1) to point to n_*matched*_ and go to next iteration. If n_*matched*_ = C_*uv*_(k^*t*^), replace p_*k*_(t) with p_*k*_(t + 1) and end the searching process.

Compared with traversal search algorithm, this method only using a small part of the SOM network, which could efficiently shorten the training time to complete and decrease the computing complexity.

**Fig 4:**
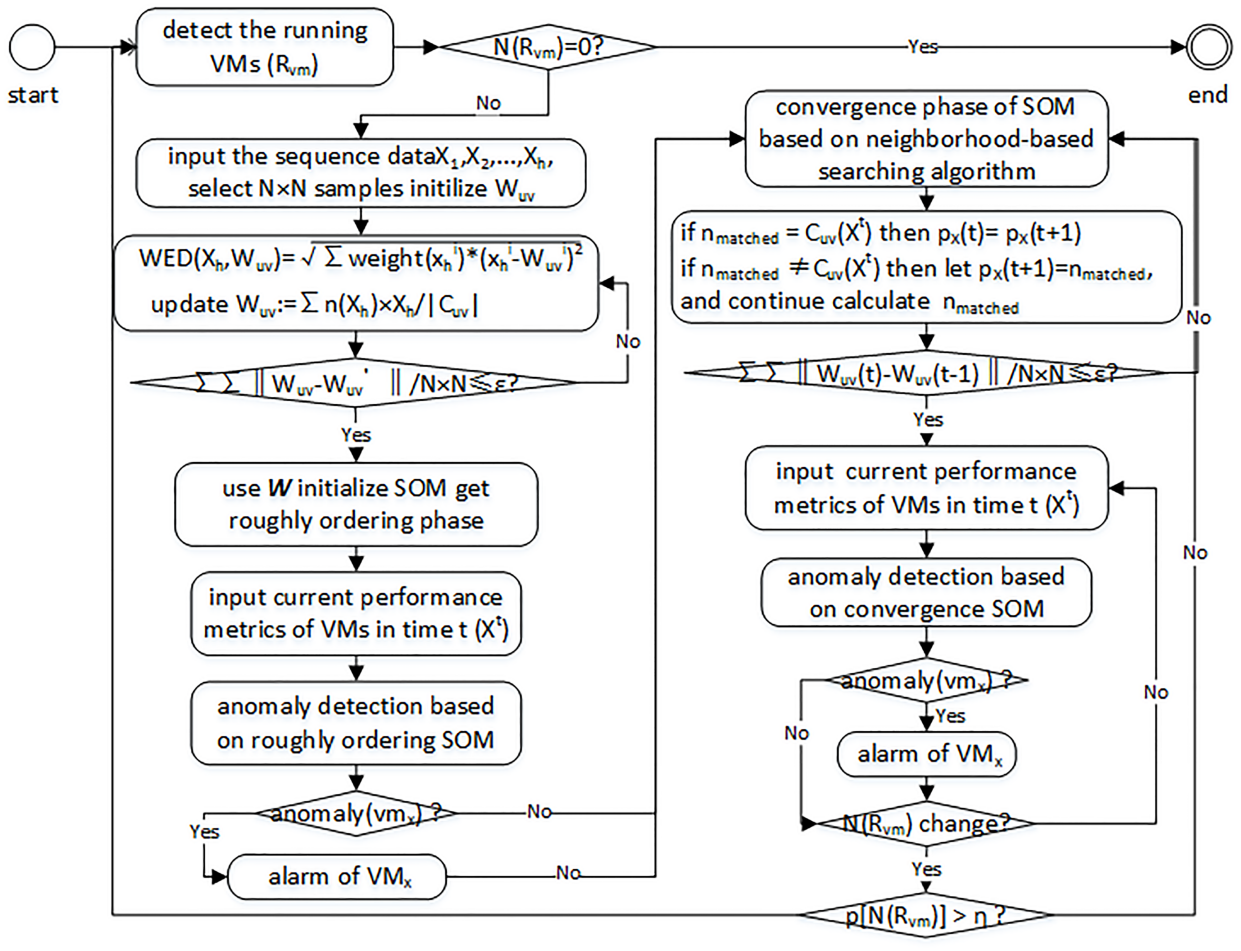
The flowchart of anomaly detection for virtual machines based on IISOM.

As stated above, IISOM is an effective anomaly detection algorithm for virtual machines on cloud platform. The IISOM detection model for virtual machines mainly consists of two parts (Fig 4). First is the iterative regress initialization phase. In this phase, the similarity of each data instance and the contributions of each dimension of data are considered and process is iterative until a predetermined minimum value *ε* is achieved. Due to the initialization process could approximately simulate the distributions of data, the roughly ordering SOM performs well in anomaly detection. Thus, when a new data instance is collected, the anomaly detection takes place in advance. If it is detected to be an anomaly, the alarm is raised. Otherwise, use it to train model until the model convergence. And the second part is the iterative convergence phase, where the neighborhoods of virtual machines are considered. The convergence process of SOM will not be retrained till the changing rate of running virtual machines exceed the threshold *η*, which is predefined to display the sensitivity to the change of virtual machines and we set it to be 0.4.

## Experiments and discussions

### Experimental environment

In this paper, the open source cloud platform OpenStack [44, 45] was used to build the experimental cloud platform with the physical servers for running virtual machines and the physical servers for running cloud management components. All the physical servers are installed the operation system CentOS6.5, while the former installs the hypervisor Xen3.2 [46], and the latter installs the cloud management components. And 100 virtual machines were deployed on this experimental cloud platform.

**Fig 5:**
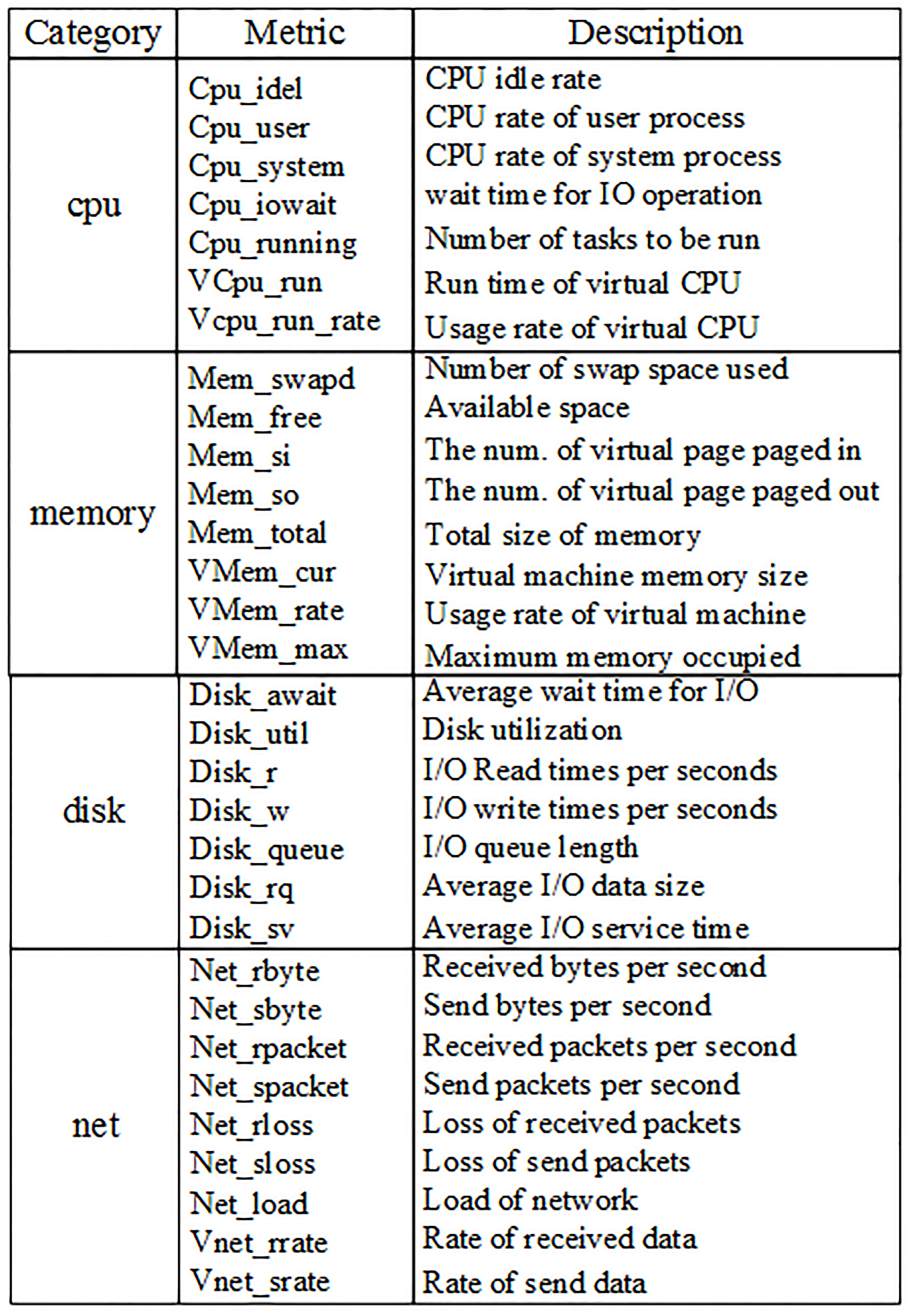
A subset of performance metrics. Four categories of virtual machines’ performance are listed.

The runtime performance metrics set of virtual machines in this platform is collected by tools such as libxenstat and libvirt [47, 48]. And during the runtime, three types of fault injection method were used to simulate system failures, that is memory leak, CPU Hog and the network Hog [49–51]. A subset of key performance metrics collected is shown in Fig 5.

### Experimental program and results

#### Experiment 1: Performance evaluate of anomaly detection for virtual machine

The performance of real-time and accuracy of IISOM was evaluated by comparing with the traditional SOM and SOMSA method.

Training: first of all, choose several virtual machines from 100 virtual machines deployed on the cloud platform, and randomly select one fault (memory leak, CPU Hog and network Hog) to inject into. Then collect 1000 runtime performance measurements from the 100 virtual machines during 10 rounds (one second per round) as the training data.

Anomaly detection: in order to estimate the performance of real-time and accuracy of the methods, one of the three faults was randomly injected into the 100 virtual machines per second and the duration time is 1 minute. The anomalies were then detected by the three trained models, traditional SOM (TSOM) model, SOMSA model and IISOM model. And the detection results were recorded.

The experimental results are shown in the following tables, Tables 1 and 2.

**Table 1:** The comparisons of TSOM, SOMSA and IISOM on the detection time.

	Injecttime(s)	Responsetime(s)	Detectiontime(s)
*TSOM*	1	2.33	77.88
*SOMSA*	1	2.21	73.76
*IISOM*	1	1.72	64.54

The fault is injected in every per second, and response time is the time the model detect the fault. The detection time is the total time during the fault injection.

**Table 2:** The comparisons of TSOM, SOMSA and IISOM on detection accuracy.

	AccuracyRate(%)
*TSOM*	≈ 97.6
*SOMSA*	≈ 98.9
*IISOM*	≈ 99.0

The Accuracy Rate is the rate of correctly detecting the anomalies during the fault injection.

It can be seen from Table 1 that compare to TSOM model and SOMSA model, the IISOM takes the shortest response time to anomalies. For IISOM, it owns to the neighborhood-based domain searching method, which controls the searching domain into a small range for every input samples then speed up the detection time. And SOMSA model achieves a better detection time than TSOM model due to the SA optimization algorithm.

Table 2 shows the comparisons of detection accuracy among the TSOM model, SOMSA model and the IISOM model, all of them could obtain a good detection accuracy. Regardless of the detection time, the SOMSA model almost has the same accuracy rate with IISOM model. However, because of the dynamic characteristics of the virtual machines on the cloud platform, a shorten detection time is necessary. So, IISOM method can have a better performance in anomaly detection of virtual machines on the cloud platform than the others.

#### Experiment 2. The effect of clustering on KDD cup

The objective of this set of experiments was to evaluate the effect of clustering on the benchmark KDD Cup [52] dataset to cluster the kind of anomalies. In KDD Cup dataset, there are 5 types of samples, the normal samples and four kinds of abnormal samples, each sample contains 41 indicators.

We select 1000 samples from the KDD Cup dataset as the training dataset, which were labeled as normal, Probe attacked, DOS attacked, U2R attacked and R2L attacked, each 200. Meanwhile, 2000 samples from KDD Cup dataset are selected as testing dataset. Two steps are carried out to evaluate these algorithms: training and detection. 1000 testing samples are used to train the detection models of IISOM, SOMSA and traditional SOM, while 2000 testing samples are used to evaluate the detection performance among these models. The results are shown in Tables 3 and 4.

**Table 3:** The experimental results of IISOM and traditional SOM on KDD cup test dataset.

Result	IISOM					TSOM				
Real label	Normal	Probe	Dos	U2R	R2L	Normal	Probe	Dos	U2R	R2L
**Normal**	376	1	8	0	15	361	4	9	2	26
**Probe**	8	379	7	0	6	17	371	9	0	3
**Dos**	16	8	546	0	2	24	5	537	2	4
**U2R**	11	0	9	192	16	13	1	7	190	17
**R2L**	13	4	2	0	381	14	7	3	0	376

Normal, Probe, Dos, U2R and R2L are five types of KDD Cup dataset.

**Table 4:** The experimental results of IISOM and SOMSA on KDD cup test dataset.

Result	IISOM					SOMSA				
Real label	Normal	Probe	Dos	U2R	R2L	Normal	Probe	Dos	U2R	R2L
**Normal**	376	1	8	0	15	370	2	7	2	19
**Probe**	8	379	7	0	6	15	375	6	1	3
**Dos**	16	8	546	0	2	24	5	537	2	4
**U2R**	11	0	9	192	16	13	1	7	192	15
**R2L**	13	4	2	0	381	10	7	3	1	379

Normal, Probe, Dos, U2R and R2L are five types of KDD Cup dataset.

According to them, we get the results of metrics in Table 5.

**Table 5:** The metrics results of IISOM, traditional SOM and SOMSA on KDD cup dataset.

Algorithm	TP	FP	TN	FN	R_*TP*_	R_*FP*_	R_*p*_	R_*accurate*_
**IISOM**	1498	24	376	102	0.936	0.060	0.984	0.937
**TSOM**	1474	41	361	126	0.921	0.102	0.973	0.917
**SOMSA**	1490	30	370	110	0.931	0.075	0.980	0.930

TP is the number of True Positive samples, FP is the number of False Positive samples, TN is the number of True Negative samples, FN is the number of False Negative samples. R_*TP*_ is the rate of TP, R_*FP*_ is the rate of FP, R_*p*_ is the rate of precision and R_*accurate*_ is the accuracy of the Algorithm.

We estimate the detection accuracy trough four parts. One is the true positive rate R_*TP*_, which describes the sensitivity of the detection algorithm to detect anomalies when an anomaly occurs. R_*FP*_ is opposite. R_*p*_ is the rate of precision, which shows the ratio of correctly identified anomalies account for all detected anomalies. R_*accurate*_ presents the proportion of true positives and false positives account for all testing samples.

#### Experiment 3. The effect of clustering on real dataset

Due to the runtime performance of virtual machine has high dynamic, experiments on the real dataset are conducted, too. 1000 running data from our cloud platform were selected as the training sample, including normal samples, CPU anomaly samples, memory anomaly samples and network anomaly samples, each 250. Similarly, experiments based on these 2000 testing samples are carried out and the results are shown in Tables 6 and 7.

**Table 6:** The experimental results of IISOM and traditional SOM on our own test dataset.

Result	IISOM				TSOM			
Real label	Normal	CPU Anomaly	Mem Anomaly	Net Anomaly	Normal	CPU Anomaly	Mem Anomaly	Net Anomaly
**Normal**	489	5	3	3	477	7	10	6
**CPUAnomaly**	2	475	11	12	6	475	8	11
**MemAnomaly**	1	14	483	2	3	9	481	7
**NetAnomaly**	9	12	8	471	10	13	11	466

CPUAnomaly, MemAnomaly and NetAnomaly are three types of abnormal performance samples collected by fault injection.

**Table 7:** The experimental results of IISOM and SOMSA on our own test dataset.

Result	IISOM				SOMSA			
Real label	Normal	CPU Anomaly	Mem Anomaly	Net Anomaly	Normal	CPU Anomaly	Mem Anomaly	Net Anomaly
**Normal**	489	5	3	3	481	8	7	4
**CPUAnomaly**	2	475	11	12	7	477	6	10
**MemAnomaly**	1	14	483	2	4	11	479	6
**NetAnomaly**	9	12	8	471	7	13	10	470

CPUAnomaly, MemAnomaly and NetAnomaly are three types of abnormal performance samples collected by fault injection.

Summarized from Tables 6 and 7, we get the metric results in Table 8, that is the true positive rate R_*TP*_ and the false positive rate R_*FP*_, the precision R_*p*_ and the accuracy rate R_*accurate*_.

**Table 8:** The metrics results of IISOM, traditional SOM and SOMSA on Real dataset.

Algorithm	TP	FP	TN	FN	R_*TP*_	R_*FP*_	R_*p*_	R_*accurate*_
**IISOM**	1918	11	489	71	0.964	0.022	0.994	0.967
**TSOM**	1899	23	477	78	0.961	0.046	0.988	0.959
**SOMSA**	1907	19	481	74	0.963	0.038	0.990	0.962

TP is the number of True Positive samples, FP is the number of False Positive samples, TN is the number of True Negative samples, FN is the number of False Negative samples. R_*TP*_ is the rate of TP, R_*FP*_ is the rate of FP, R_*p*_ is the rate of precision and R_*accurate*_ is the accuracy of the Algorithm.

It can be found from results (Tables 5 and 8) that IISOM has the highest accuracy followed with SOMSA and traditional SOM. IISOM leverages heuristic-based initialization algorithm rather than random initialization used in traditional SOM and SOMSA algorithms, which makes it have a fast learning ability for a runtime performance and detect in time. Though SOMSA uses simulated annealing algorithm to optimize the excited neuron’s weight and its neighborhoods to improve the accuracy, it takes a longer time to train the self-organizing map network by randomly selecting adjacent center neurons to replace the current center neurons. So SOMSA does not work well on anomaly detections for virtual machines with high dynamic. Besides, IISOM algorithm takes into account that the contributions of each dimension to self-organizing map network is different. If each dimension in the input space is treated equally, just like traditional SOM algorithm and SOMSA algorithm, it may lead to the imbalance of self-organizing map network, in which a great change in some dimensions cannot cause the attention of the SOM and a subtle change in some dimensions may result in a serious fluctuation. Weighted Euclidean Distance algorithm uses different weight values to calculate the similarity (Euclidean Distance) between the input space (input samples) and the output space (neurons) to balance the contribution and improve the quality of the trained SOM model. These two experiments (based on KDD Cup dataset and the real dataset) approved the result that IISOM has a higher accuracy than traditional SOM and SOMSA algorithm, improving the true positive rate and lowing the false positive rate.

#### Experiment 4. Estimate the performance of models

Although IISOM performs better in the known attacks or anomalies in the same testing samples than the other algorithms, there may be of inaccuracy for unknown attacks or anomalies. So, we conduct an experiment on scalability to verify whether IISOM could detect unknown attacks or anomalies better than traditional SOM and SOMSA or not. The results of three SOMs are shown in Table 9.

**Table 9:** The experimental results of IISOM and traditional SOM on our own test dataset.

Performance Algorithm	Dos		U2R		Known Anomalies	Unknown Anomalies
	R_*TP*_	R_*FP*_	R_*TP*_	R_*FP*_		
**IISOM**	0.932	0.015	0.887	0.019	0.953	0.941
**TSOM**	0.806	0.031	0.695	0.018	0.912	0.7783
**SOMSA**	0.854	0.011	0.793	0.023	0.932	0.866

Dos and U2R are two attacked samples. Table shows the accuracy of detecting Known Anomalies and unknown anomalies, the rate of false positive and rate of true positive.

Two types of attacked samples are selected, in which DOS has the maximum number in the whole KDD Cup testing dataset while U2R (User-to-Root) is the minimum. Using the SOM model we trained in Experiment 2 to test these two kinds of attacked samples. From Table 9, it can be interpreted that IISOM algorithm has a higher true positive rate than traditional SOM and SOMSA. In addition, we compare the accuracy rate of known anomalies detecting with unknown anomalies detecting to evaluate the quality of models. After training and testing, it can be seen from Table 9 that although the false positive rate of IISOM is relatively higher than SOMSA on DOS attacked samples, it has an obvious advantage in unknown anomalies detection than SOMSA. Furthermore, it can be found that whether the intrusion is large or not, IISOM algorithm performance as well as usual.

**Fig 6:**
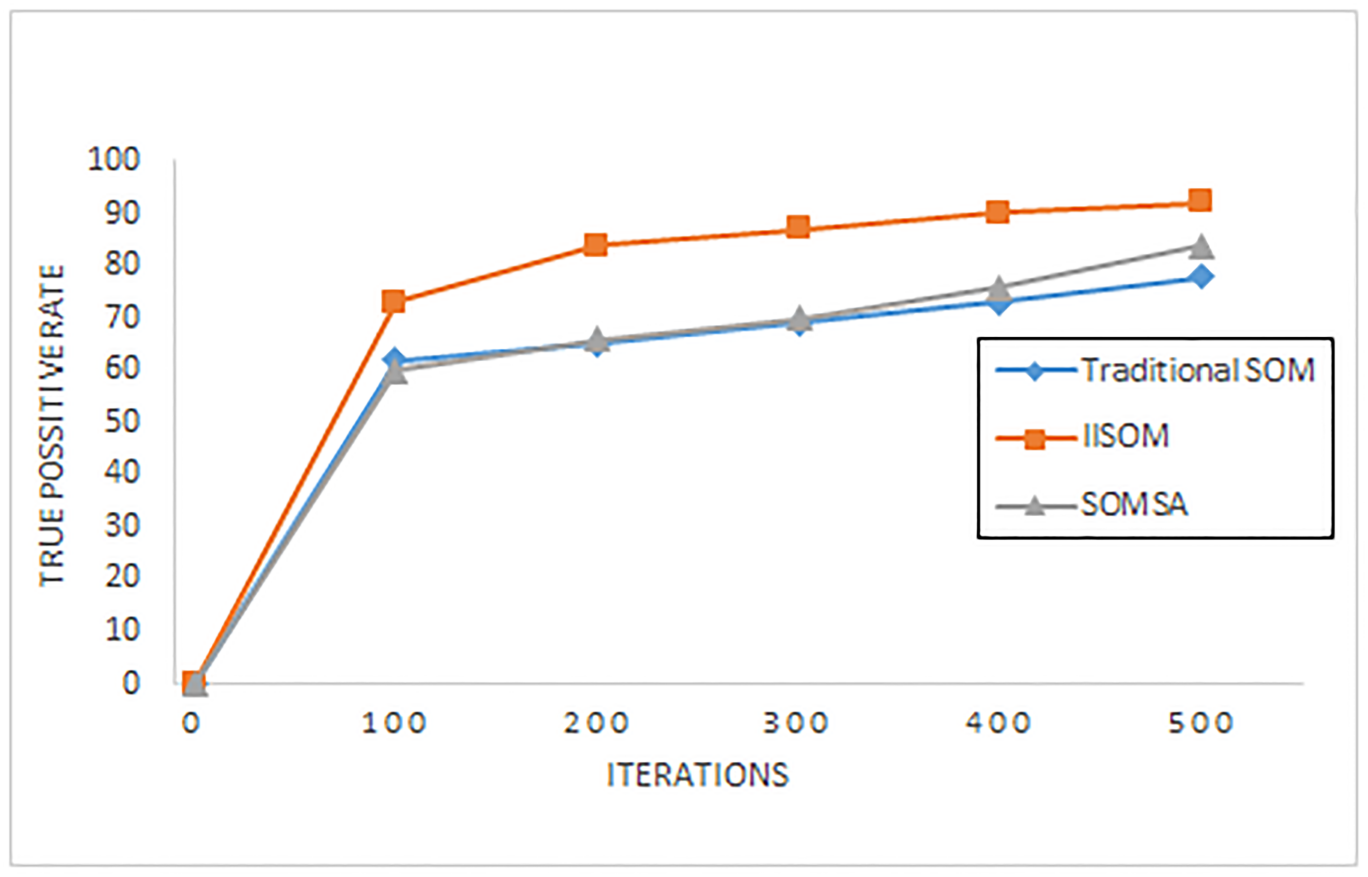
The true positive rate for IISOM, traditional SOM and SOMSA with different iterations.

**Fig 7:**
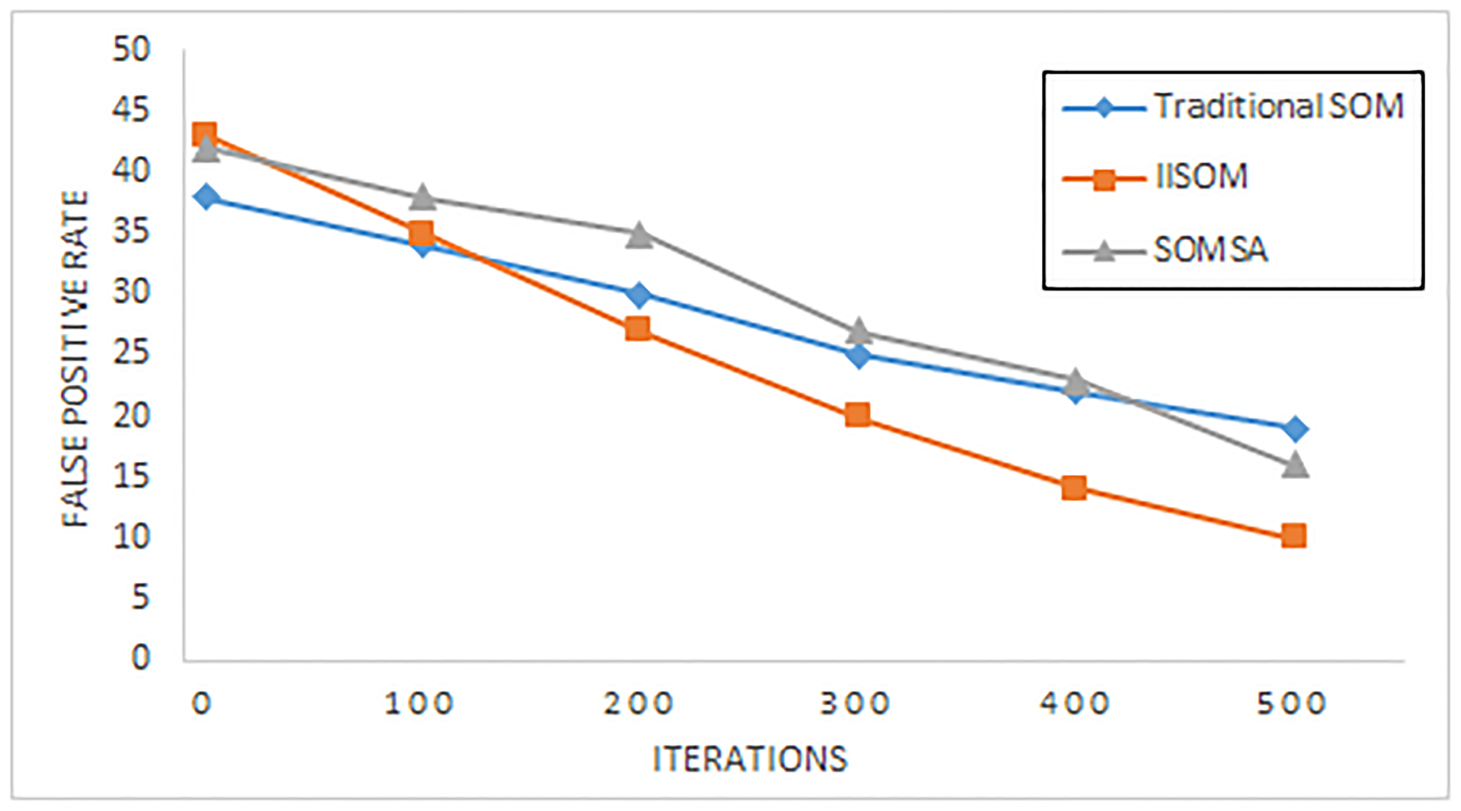
The false positive rate for IISOM, traditional SOM and SOMSA with different iterations.

In order to embody the advantage of the IISOM algorithm, two figures are used to reflect the true positive rate R_*TP*_ and the false positive rate R_*FP*_. Fig 6 shows the true positive rate of the IISOM, traditional SOM and SOMSA with different iterations. Fig 7 shows the false positive rate of the IISOM, traditional SOM and SOMSA with different iterations.

From these two figures, it can be seen that for all the three SOM algorithms, accuracy rate keeps rising with the increase of iteration, while the false rate reduces. For IISOM algorithm, it has a relatively higher true positive rate and a lower false positive rate than the other two algorithms at the same number of iterations. For SOMSA, when the iterations is lower than 400, both true positive rate and false positive rate are relatively smooth, while as the number of iterations continues to increase, the accuracy rate is improved significantly than the traditional SOM.

#### Experiment 5. Estimate the parameters

The main advantage of the IISOM method is that it could balance the contribution of each dimension in the input space, rapidly and automatically search the Best Matching Neurons (BMN) by competition, avoiding giving any prior knowledge. However, several parameters should be tuned in order to obtain the fitness and smoothness SOM network. Here, we study the performance of IISOM by varying one parameter at a time.

**Fig 8:**
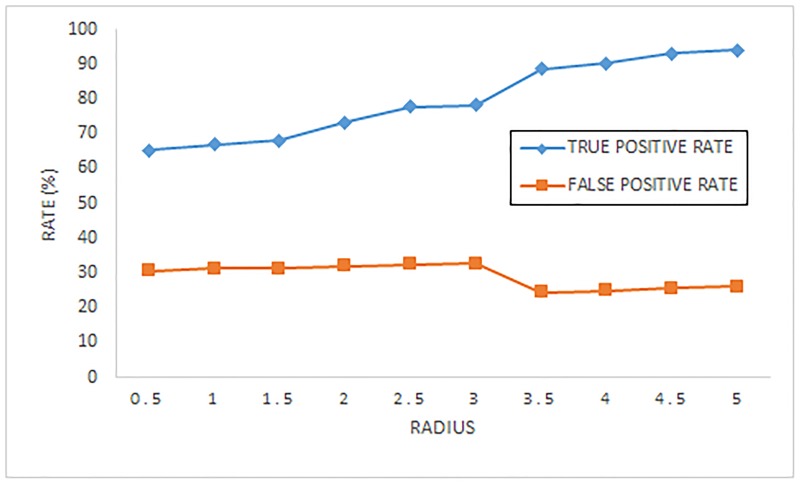
The performance for IISOM by varying the initial neighborhood size(radius).

**Fig 9:**
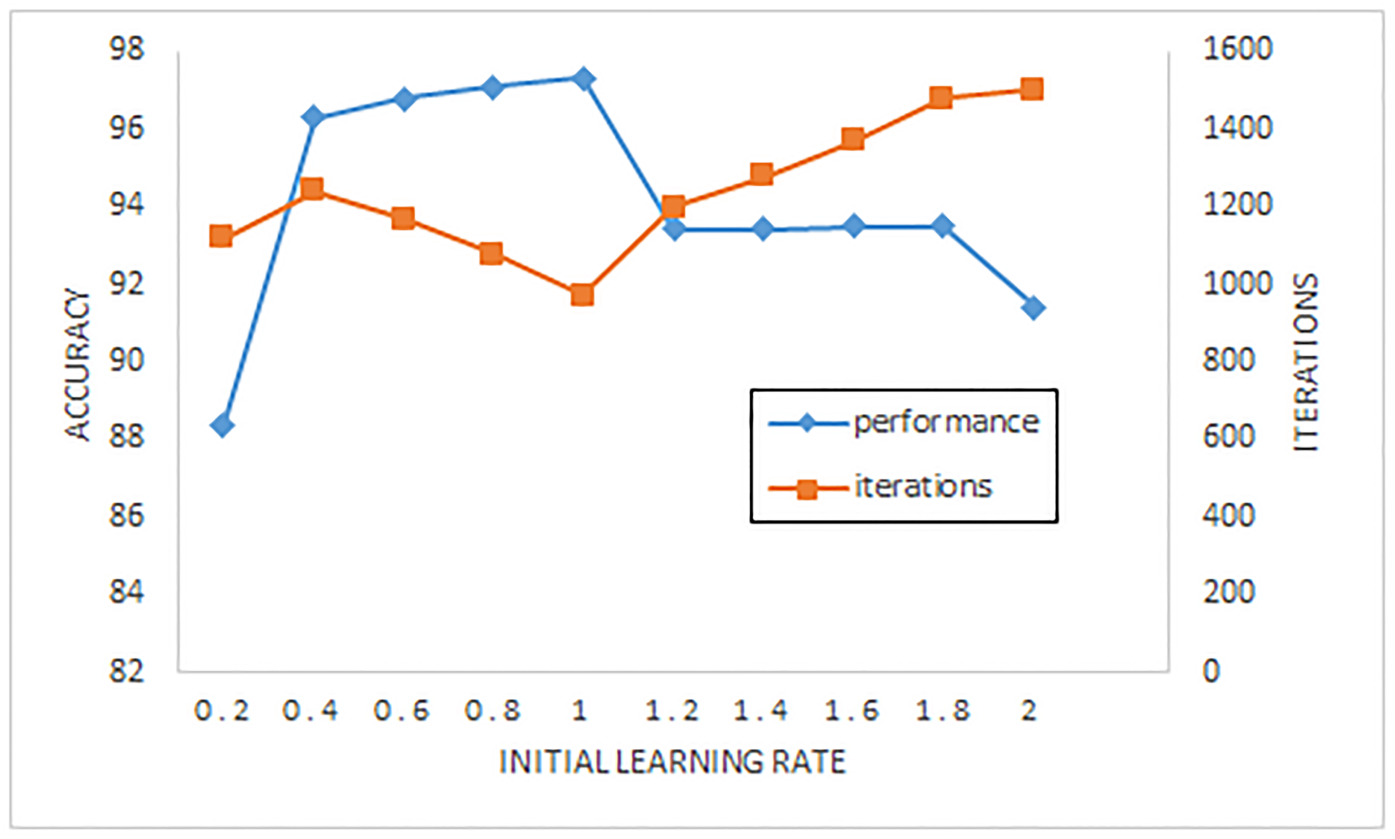
The performance for IISOM by varying the initial learning rate.

The parameters we discuss about are the map size of SOM k, the neighborhood size *δ* and the learning rate *γ*. In order to illustrate their influence on the detection, the Table 10 and following Figs (Figs 8 and 9) display the impact on the quality and accuracy of IISOM.

**Table 10:** The impact of map size of SOM on the quality of IISOM.

Map size of SOM	QE	TE
5 × 5	0.12	0.01
10 × 10	0.11	0.02
15 × 15	0.11	0.01
18 × 18	0.10	0.02
21 × 21	0.09	0.02

QE is the quantzation error, TE is the topographic error.

As we can see from Table 10, two types of quantities are used to evaluate the quality of the SOM that is quantization error (QE) and topographic error (TE) [53]. QE is the average distance between each input vector and its cluster center, the smaller the distance, the higher the detection accuracy. TE is the proportion of all input vectors for which first and second cluster centers are not adjacent, a smaller value comes to a smooth model. IISOM automatically balances the influence of each dimension in the input space by weighted Euclidean distance algorithm, which could maintain the topology preservation and reduce the distance between each input samples with its cluster center. Combined with the experimental results, it can be found that the map size of SOM has a few influence on the quality of the IISOM when the initial size of training neighborhood (radius) and learning rate are set to be 3.5 and 1, respectively.

To evaluate the neighborhood size (radius) and learning rate well, the map size of SOM is set to be 15 × 15. Fig 8 displays the true positive rate and false positive rate of IISOM with different initial neighborhood size (radius), in which the initial learning rate is set to be 1. Both true positive rate and false positive rate achieve a better value around the radius 3.5, greater than or less than the value the accuracy rate is low. Fig 9 illustrates the performance of IISOM in terms of the number of iterations and the accuracy rate with different initial learning rate. From this figure we can see that the initial learning rate is related to the necessary iterations for convergence, a smaller or larger value will lead to more iterations. In the training process of IISOM, the learning rate is monotone decreasing during the iteration, therefore, it is efficient to choose a good initial learning rate. A small initial learning rate makes a small contribution of each sample to train the SOM network, which causes under fitting the status of testing data to decline the accuracy rate. Vice versa, a large one leads to over fitting, decreasing the convergence velocity. As shown in Fig 9, when the initial learning is 1, the accuracy rate achieves the highest value and the iterations is lowest, in which the initial neighborhood size is set to be 3.5.

### Discussion

Cloud platforms with characteristics of resource sharing, allocation in demand and virtualization appear more and more users to lease resources in pay-as-you-go fashion and deploy their own systems on it to improve the utilization of hardware and software resources and reduce the cost. However, due to the ever-growing complexity and dynamic of cloud computing systems, it is susceptible to resource contentions, software bugs, hardware failures or administrators’ mistakes, which can significantly affect the system performance. In order to avoid the performance anomaly, a number of anomaly detection techniques [54–57] were proposed to proactively and reactively detect anomalies, but due to their detection mechanisms, they mostly lack scalability and often require prior knowledge, thus making them unsuitable for virtual machines detection on cloud platforms. Thus, we propose the Improved Incremental SOM (IISOM) algorithm to accelerate detecting process and improve the quality of detection model by considering the virtual machines’ high dynamic and complexity.

#### Strengths and limitations

From the experimental results, it can be seen that IISOM is superior to the traditional SOM and SOMSA algorithm for anomaly detection of virtual machines. With the help of heuristic-based initialization method and neighborhood-based searching method, IISOM greatly enhances the detection accuracy and the detection rate. Besides, by considering the contribution of each dimension of the performance metrics, the IISOM algorithm outperforms the traditional SOM and SOMSA algorithm, achieving a highest quality model than the others.

There are nonetheless several limitations of the current IISOM method. The parameters of map size of SOM, learning rate and neighborhood size still have to be predefined by empirical, and the current heuristic-based initialization method takes on the opinion that the initial weight value of neurons would approximately reflect the data distribution. But the data distribution is usually unknown in advance, several iterative regression computations have to be done during the initialization.

#### Future work

Anomaly detection for virtual machines on cloud platforms has a high demand of algorithm for detection accuracy, self adaption and real-time capability. As the lack of adaptive map size, a data-driven density-based detection method will be introduced into the IISOM to improve the detection model. In addition, with the growing scale of cloud platform, it will be increasingly subjected to novel attacks and other anomalies. Thus, incorporating some novelty detection method into this IISOM method is a promising future direction. What’s more, to enhance the technology and keep up with the trends, we plan to apply this IISOM to some new fields, such as mobile Augmented Reality [58], Mobile Landmark Recognition [59], Mobile Visual Location Recognition [60], Image processing [61, 62] and so on, to deal with the high dimensional data and meet the demands of real-time capability.

## Conclusion

Considering with the feature of complexity and high dynamicity of cloud platform, the Improved Incremental SOM (IISOM) algorithm is proposed to identify and predict anomalies to keep the platform dependable. Weighted Euclidean Distance (WED) method and a heuristic-based initial method are incorporated into the Self-organizing Map (SOM) algorithm to reduce computational overhead, shorten the training time and self-adaptation in dynamic environment. To evaluate the proposed algorithm, five experiments are performed. Experiment 1 is carried out to evaluate the real-time property and detection accuracy of the IISOM compared to SOMSA and TSOM. The different detection models based on KDD Cup dataset and real dataset are trained through experiments 2 and 3 to evaluate the performance. Experiment 4 is set to estimate the quality or performance of the models when encounters unknown anomalies. Compared with traditional SOM and SOMSA on the four experiments, it shows that IISOM has more advantages in detecting accuracy, convergence velocity, and performance even though the anomalies are large and unknown. For further analysis of IISOM algorithm, several important parameters are considered in Experiment 5. From which, it can be summarized that the map size of SOM k has few influence in the model training process due to the dimensional balance based on WED algorithm, while the initial neighborhood size *δ* and the initial learning rate *γ* are sensitive to the model training. It can be found that the model performs well when *δ* is 3.5 and *γ* is 1.
